# Oxygenation Inhibits the Physiological Tissue-Protecting Mechanism and Thereby Exacerbates Acute Inflammatory Lung Injury

**DOI:** 10.1371/journal.pbio.0030174

**Published:** 2005-05-03

**Authors:** Manfred Thiel, Alexander Chouker, Akio Ohta, Edward Jackson, Charles Caldwell, Patrick Smith, Dmitry Lukashev, Iris Bittmann, Michail V Sitkovsky

**Affiliations:** **1**Laboratory of Immunology, National Institute of Allergy and Infectious DiseasesNational Institutes of Health, Bethesda, MarylandUnited States of America; **2**Clinic of AnaesthesiologyUniversity of MunichGermany; **3**New England Inflammation and Tissue Protection Institute, Northeastern UniversityBoston, MassachusettsUnited States of America; **4**Pharmacology/Medicine Pittsburgh, University of Pittsburgh School of MedicinePennsylvaniaUnited States of America; **5**Pathology, Klinikum GrosshadernUniversity of MunichGermany; Royal Infirmary EdinburghUnited Kingdom

## Abstract

Acute respiratory distress syndrome (ARDS) usually requires symptomatic supportive therapy by intubation and mechanical ventilation with the supplemental use of high oxygen concentrations. Although oxygen therapy represents a life-saving measure, the recent discovery of a critical tissue-protecting mechanism predicts that administration of oxygen to ARDS patients with uncontrolled pulmonary inflammation also may have dangerous side effects. Oxygenation may weaken the local tissue hypoxia-driven and adenosine A_2A_ receptor (A_2A_R)-mediated anti-inflammatory mechanism and thereby further exacerbate lung injury. Here we report experiments with wild-type and adenosine A_2A_R-deficient mice that confirm the predicted effects of oxygen. These results also suggest the possibility of iatrogenic exacerbation of acute lung injury upon oxygen administration due to the oxygenation-associated elimination of A_2A_R-mediated lung tissue-protecting pathway. We show that this potential complication of clinically widely used oxygenation procedures could be completely prevented by intratracheal injection of a selective A_2A_R agonist to compensate for the oxygenation-related loss of the lung tissue-protecting endogenous adenosine. The identification of a major iatrogenic complication of oxygen therapy in conditions of acute lung inflammation attracts attention to the need for clinical and epidemiological studies of ARDS patients who require oxygen therapy. It is proposed that oxygen therapy in patients with ARDS and other causes of lung inflammation should be combined with anti-inflammatory measures, e.g., with inhalative application of A_2A_R agonists. The reported observations may also answer the long-standing question as to why the lungs are the most susceptible to inflammatory injury and why lung failure usually precedes multiple organ failure.

## Abstract 2

A mouse model suggests that oxygen therapy may exacerbate lung injury by weakening the anti-inflammatory mechanisms driven by hypoxia.

## Introduction

Many clinical conditions, including aspiration, trauma, and hemorrhagic shock, are frequently followed by pulmonary and systemic infectious and septic complications that lead to pulmonary dysfunction and subsequent lung failure. Acute lung injury or its more severe form, the acute respiratory distress syndrome (ARDS), occur with a frequency of approximately 130.000 cases and more than 50.000 deaths from ARDS per year in the United States alone [1]. Intubation with mechanical ventilation represent one of the most widely used prophylactic and therapeutic clinical interventions to counteract the insufficient pulmonary oxygen-delivering capacity in patients who suffer from severe lung inflammation. Although the majority of patients respond well to oxygen therapy, and oxygen toxicity is an uncommon occurrence in intensive care medicine [2], there remains the possibility that oxygen therapy may be suboptimal in ARDS patients, as it may promote deleterious pulmonary inflammation, which fuels this disease process. Since the magnitude and duration of lung inflammation has been shown to determine the final outcome of ARDS patients [3], it is important to carefully evaluate the possible adverse effects of oxygen on inflammatory processes.

We assumed that lung tissues are protected from overactive immune cells by the same hypoxia-driven mechanism and immunosuppressive adenosine A_2A_ receptor (A_2A_R)-mediated mechanism that was recently shown to play a critical role in the down-regulation of inflammation and tissue damage in different models [4–7]. Accordingly, it is likely that bacterial toxin-activated immune cells (e.g., granulocytes) cause collateral lung tissue damage with impairment of the local microcirculation and blood supply, thereby contributing to the pathogenesis of acute lung injury. The ensuing tissue damage-associated hypoxia facilitates the accumulation of extracellular adenosine [8–12], which then triggers the activation of immunosuppressive A_2A_Rs on activated immune cells and causes the accumulation of immunosuppressive intracellular cyclic adenosine 3′,5′-monophosphate (cAMP). This cAMP, in turn, inhibits signaling pathways that are required for synthesis and secretion of proinflammatory and cytotoxic mediators by immune cells, and thereby protects remaining healthy tissues from continuing immune damage. Since this physiological tissue-protecting mechanism depends on the hypoxia-produced extracellular adenosine [8–12], and since the oxygenation of lungs in intubated patients is performed to increase oxygen tension—with the goal of abolishing the hypoxia but disrupting the adenosine accumulation—we reasoned that such interruption of the hypoxia → adenosine → A_2A_R pathway by oxygenation could lead to a disengagement of the critical tissue-protecting mechanism and to an unintended exaggeration of inflammatory lung damage (iatrogenic disease). Thus, oxygenation may eliminate this lung-protecting pathway and thereby contribute to pulmonary complications.

We confirm our prediction in several in-vivo models of lung infection and inflammation and report that oxygenation does indeed strongly exacerbate inflammatory lung damage and accelerate death in mice by the interruption of the hypoxia → adenosine → A_2A_R pathway. We also suggest an effective and feasible therapeutic countermeasure to prevent deleterious effects of oxygenation: Exogenously added synthetic A_2A_R agonist compensated for the loss of endogenously formed adenosine in inflamed lungs of oxygenated mice, and thereby prevented inflammatory lung injury and prevented death.

## Results

### Exacerbation of Inflammatory Lung Injury and Death in Oxygenated Mice

To test our prediction, we subjected mice to inhalation of combined toxins from gram-positive and gram-negative bacteria. In this model of polymicrobial lung infection, intratracheal (IT) injection of both lipopolysaccharide (LPS) and staphylococcal enterotoxin B (SEB) strongly potentiates their toxicity [13].

The results of these assays confirmed the prediction of exaggerated lung injury in mice in conditions that mimic therapeutic oxygenation, and this is reflected in the dramatic increases in inflammatory lung damage in different in vivo and ex vivo assays (Figures 1 and 2).

**Figure 1:**
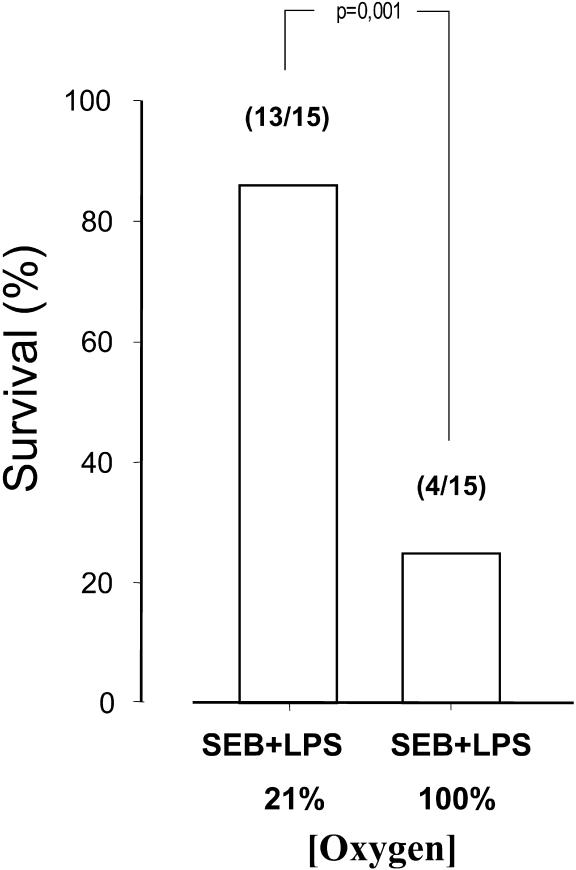
Increased Death Rate upon Oxygenation of Mice with Acute Lung Injury Mice were IT injected with SEB and LPS to model polymicrobial infection and were exposed to 21% or 100% oxygen for 48–60 h. Determination of time-dependent survival curves was prohibited by considerations of unrelieved severe respiratory distress in NIH-approved animal care protocol, which required termination of experiments immediately after differences between groups became apparent. Major differences between groups occurred within 48–60 hours after IT injection of toxins, when the majority of oxygenated animals with inflamed lungs had died, while the nonoxygenated, obviously sick control mice with inflamed lungs were still alive.

**Figure 2:**
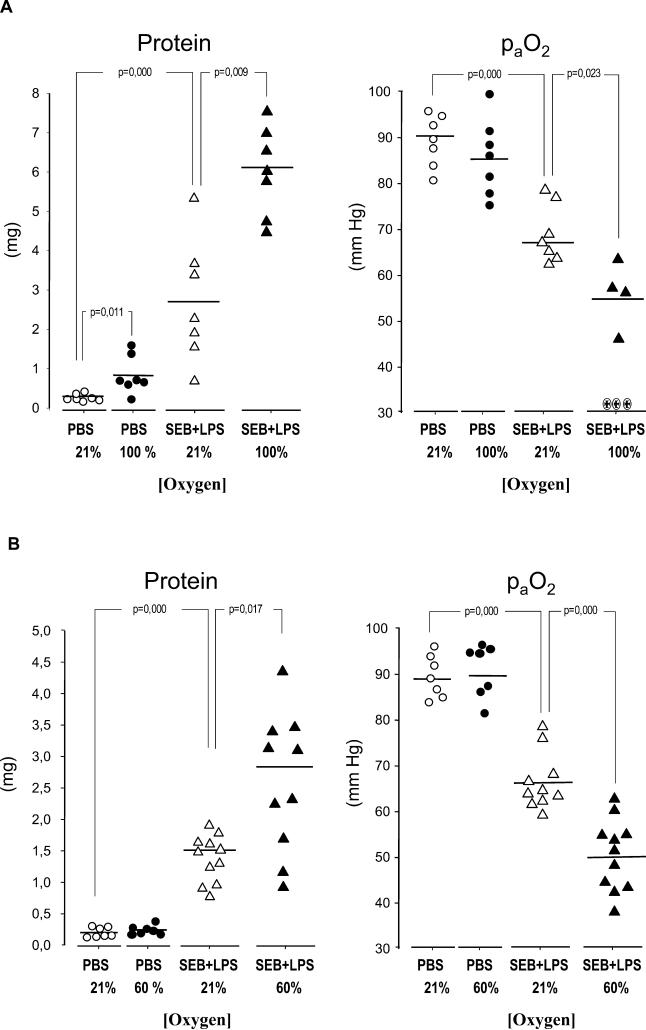
Exacerbation of Inflammatory Lung Injury after Exposure of Mice to Different Concentrations of Oxygen (A) Enhanced lung vascular permeability (left graph) and impairment of lung gas exchange (right graph) in mice breathing 100% O_2_ upon induction of acute lung injury. Following IT injection of mice with SEB and LPS, animals breathed 21% or 100% oxygen. After 48 h, lung vascular permeability and lung gas exchange were determined by the amount of protein recovered by BAL or by measuring pO_2_ values in arterial blood drawn, respectively, 15 min after return of mice to normal atmosphere. During this equilibration period, three out of seven mice previously exposed to 100% oxygen died, so that no arterial blood gas analyses could be performed, but BAL protein concentrations were determined immediately thereafter. (B) Increased lung vascular permeability (left graph) and impairment of lung gas exchange (right graph) in mice with acute lung injury even upon exposure to lower levels of oxygen (60%), which are considered clinically safe in humans. Experimental conditions were the same as in (A), except oxygen concentration was 60% instead of 100%.

Five times more mice with inflamed lungs died after exposure to 100% oxygen than those left at 21% ambient oxygen tension (Figure 1). This was further confirmed by a much more pronounced increase in the alveolocapillary permeability and severe overall impairment of lung gas exchange, as evidenced by the increase in the amount of protein recovered from the alveolar space by bronchoalveolar lavage (BAL), as well as by the decrease in arterial oxygen partial pressure (pO_2_) values of previously oxygen-exposed mice when returned to normal atmosphere (Figure 2A). Although exposure of mice to 100% oxygen alone (with no toxin inhalation) did induce a small accumulation of BAL fluid protein, the magnitude of that effect could not account for the dramatic increases in lung vascular permeability and impairment of lung gas exchange when both toxins and oxygenation were used (Figure 2A).

The exacerbation of inflammatory lung injury was also observed when mice were exposed to 60% oxygen, a concentration considered in human patients to be fairly safe. Compared to toxin-injected animals breathing 21% oxygen, those breathing 60% oxygen accumulated much more exudate proteins in the alveolar spaces and exhibited impaired arterial blood oxygen tensions (Figure 2B). Exposure of toxin-injected mice to 60% oxygen, however, did not result in death in the short-term assays we used.

These observations confirmed the prediction that inflamed lung injury is exacerbated by oxygenation; however, the development of therapeutic countermeasures requires testing of the validity of our underlying assumptions and conclusive identification of the molecular mechanisms of these proinflammatory effects of oxygen.

### Oxygenation Disrupts the Hypoxia → A_2A_R-Mediated Pathway

The observed effects of oxygenation could not be accounted for by direct toxic effects of oxygen, since these effects of oxygen take much longer to manifest [2, 14, 15] and therefore are unlikely to fully account for the dramatic lung injury observed in our short-term experiments (Figures 1 and 2).

To test whether oxygen enhances inflammatory tissue damage by eliminating the hypoxia → adenosine → A_2A_R pathway, which is hypothesized as being responsible for protecting inflamed lungs and down-regulating neutrophils, we (i) determined the role of neutrophils in inflammatory lung injury, (ii) tested for A_2A_R expression on these cells to demonstrate their susceptibility to modulation by endogenously produced adenosine, (iii) analyzed the degree of inflammatory lung tissue damage in *A_2A_R* gene-deficient mice or in wild-type mice treated with highly selective pharmacologic antagonists for A_2A_Rs, and (iv) tested whether the hypoxia has a lung-protective role and whether there is a direct linkage between hypoxia (upstream) and A_2A_Rs (downstream) in the proposed lung-protective hypoxia → A_2A_R pathway.

To diminish the suffering of animals with severely inflamed and damaged lungs, in the experiments described in the next section we used the less-severe lung injury model established by the injection of LPS alone instead of the polymicrobial (SEB + LPS) model of lung damage.

### Neutrophils Are Involved in Inflammatory Lung Injury and Are Inhibited by A_2A_Rs

In agreement with an important pathogenic role for granulocytes in acute lung injury [16, 17], we confirmed that inflammatory lung injury elicited by IT injection of LPS in wild-type mice is in large part mediated by granulocytes, because the depletion of polymorphonuclear leukocytes (PMNs) with the anti-Gr-1 monoclonal antibody resulted not only in a substantial decrease in the number of cells in the BAL fluid but also in a much less pronounced endotoxin-induced lung injury (Figure 3A). This finding was further supported by the correlation between the number of PMNs and the degree of lung vascular permeability as reflected by the amount of protein recovered from the alveolar space by BAL (Figure 3B).

**Figure 3:**
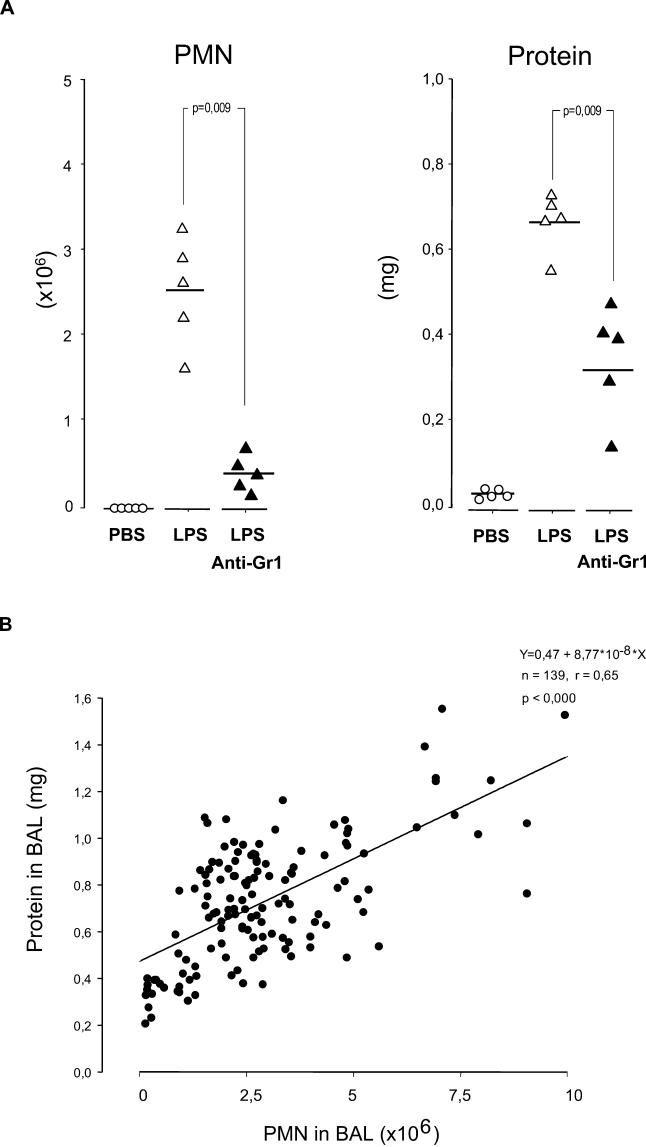
Role of PMNs in the Pathogenesis of LPS-Induced Lung Injury (A) Depletion of granulocytes attenuates the endotoxin induced rise in alveolocapillary permeability. Pretreatment of mice with anti-Gr-1 was followed by a significant decrease in the number of granulocytes (left graph) and a significant reduction of the total amount of protein (right graph) recovered by BAL 48 h after IT LPS injection. (B) The more granulocytes immigrated into the alveolar spaces, the higher the alveolocapillary permeability rose. Bivariate analysis according to Pearson revealed a statistically significant correlation (*p* < 0,001) between the number of PMNs and the amount of protein in the BAL fluid 48 h after IT LPS injection, suggesting that inflammatory lung injury after IT injection of LPS is mostly mediated by granulocytes.

To answer the question of whether murine neutrophils are susceptible to inhibitory effects of endogenously produced adenosine via A_2A_R signaling, we characterized the effects of increasing concentrations of the highly specific A_2A_R agonist CGS21680 on the oxygen radical production of granulocytes taken from the blood of healthy mice. Surprisingly, respiratory burst activity of such “naïve” granulocytes was only poorly inhibited by A_2A_R signaling (Figure 4A, left graph). Therefore it was important to test whether “in vivo-activated” granulocytes isolated from inflamed lungs of IT LPS-injected mice might have up-regulated their A_2A_R expression. As expected, significant up-regulation of A_2A_R was observed in experiments in which CGS21680 greatly enhanced the chemotactic peptide N-formyl-methionyl-leucyl-phenylalanine (fMLP)-stimulated cAMP production of in vivo-activated neutrophils isolated from inflamed lungs from wild-type mice, but not of naïve granulocytes obtained from the bone marrow of healthy wild-type control mice (Figure 4B, left graph). In a genetic control, the cAMP increase was absent in in vivo-activated granulocytes from *A_2A_R* gene-deficient mice (Figure 4B, right graph). In addition, the adenosine-induced cAMP increase was inhibited in wild-type granulocytes by the selective antagonist of A_2A_Rs ZM241385 (Figure 4B, right graph). Results of parallel assays confirmed increased expression of A_2A_R on activated granulocytes, since CGS21680 inhibited the functional response of in vivo-activated granulocytes (i.e., as evidenced by the production of tissue-damaging reactive oxygen species) much more strongly than in naïve cells (Figure 4A*,* right graph).

**Figure 4:**
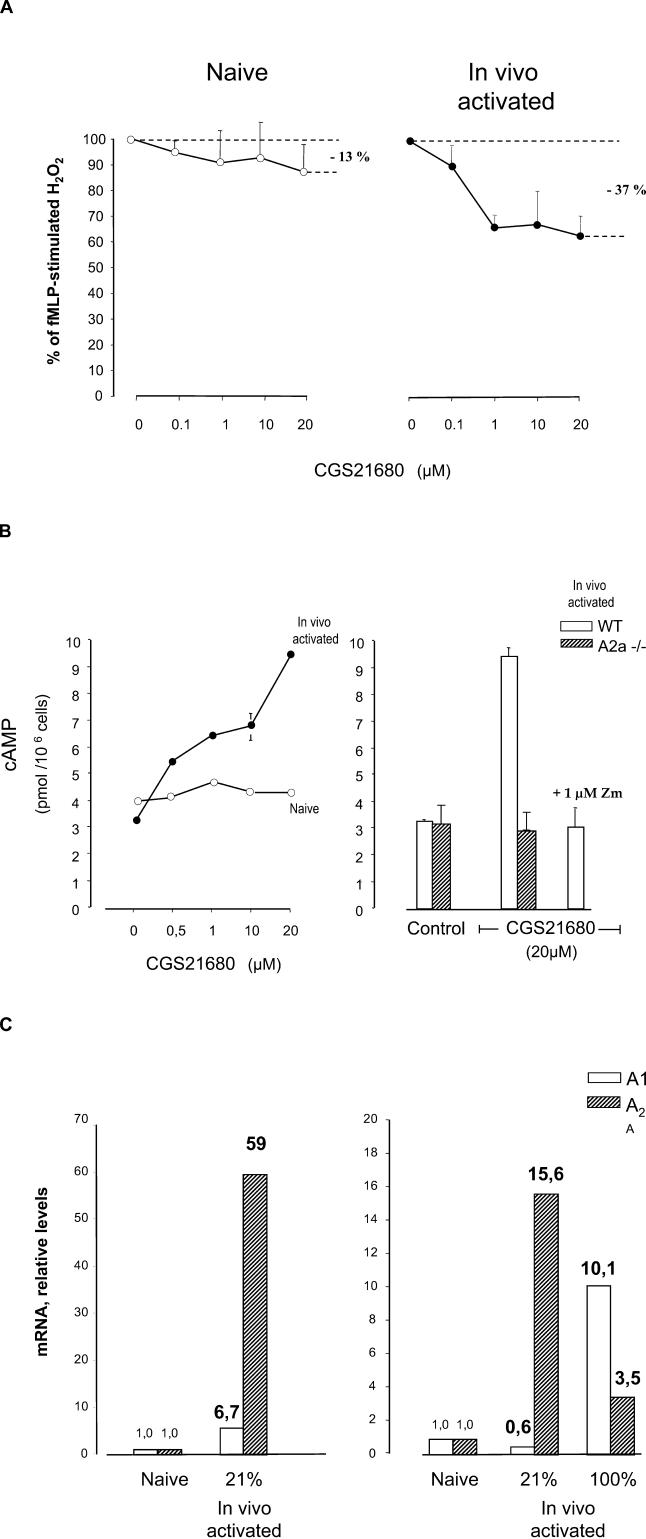
Evidence for the Up-Regulation of Immunosuppressive A_2A_R Expression on In Vivo-Activated Granulocytes Isolated from Inflamed Lungs (A) The selective A_2A_R agonist CGS21680 inhibited the fMLP-stimulated hydrogen peroxide production by granulocytes in blood of healthy mice to only a small degree, reflecting low levels of expression of A_2A_R on naïve blood granulocytes. In contrast, granulocytes recovered by BAL from inflamed lungs 48 h after IT LPS injection were much more inhibited by CGS21680, demonstrating functional up-regulation of A_2A_R on in vivo-activated cells. (B) CGS21680 induces cAMP accumulation in in vivo-activated granulocytes isolated from lungs 48 h after IT LPS injection. No effects of the A_2A_R agonist were observed in naïve granulocytes obtained from bone marrow of healthy mice (left graph) or in in vivo-activated granulocytes recovered from inflamed lungs of *A_2A_R* gene-deficient mice (right graph). The CGS21680-stimulated cAMP production observed in lung granulocytes obtained from wild-type mice could also be antagonized by the selective A_2A_R antagonist ZM241385. Naïve bone marrow granulocytes were used for cAMP measurements, since it was impossible to isolate naïve cells from blood of healthy mice in sufficient numbers. (C) Higher levels of A_2A_R-specific mRNA in in vivo-activated granulocytes. In parallel with the much stronger A_2A_R agonist-induced inhibition of hydrogen peroxide production and accumulation of cAMP in in vivo-activated granulocytes, the relative levels of A_2A_R-specific mRNA were much higher in in vivo-activated granulocytes obtained from inflamed lungs 48 h after IT LPS injection, as compared with naïve granulocytes isolated from the bone marrow of healthy mice (left graph). Up-regulation of A_2A_R mRNA in in vivo-activated granulocytes was confirmed in another set of experimental animals breathing 21% oxygen, but was increased to a much lesser extent in animals subjected to 100% oxygen (right graph). Levels of A_1_R mRNA did not change much in inflammatory lung granulocytes from animals breathing normal atmosphere, but were clearly increased in mice exposed to 100% O_2_. In the two sets of experiments (left and right graphs), granulocytes were pooled from five and six mice per treatment, respectively. Taken together, the results demonstrate that granulocytes recovered from alveolar spaces of inflamed lungs did, indeed, up-regulate their A_2A_R expression during these in vivo lung injury assays, thereby confirming and extending previous findings in other inflammation models [11, 53]

The results of mRNA expression studies were in agreement with above functional studies of adenosine receptors, and a much stronger increase in A_2A_R mRNA levels was observed in in vivo-activated PMNs than in naïve cells, with only small changes in A_1_ receptor mRNA expression in the first experiment (Figure 4C, left graph). These findings were further confirmed in a second experiment in which the effect of 100% oxygen was compared to that of normal atmosphere (Figure 4C, right graph). When compared to the several-fold increase in A_2A_R mRNA expression levels in inflammatory granulocytes from mice exposed to 21% oxygen, these levels were up-regulated to a much lesser extent in mice breathing 100% oxygen. As in the first experiment, no changes were observed in A_3_ mRNA expression, while expression of A_2B_ receptors showed changes similar to those of A_2A_R mRNA (unpublished data). Interestingly, the expression of A_1_ mRNA levels was increased in inflammatory lung granulocytes obtained from hyperoxic animals. Thus, the decreased up-regulation of A_2A_R-specific mRNA that was observed in parallel with an increased A_1_ receptor-specific mRNA expression in inflammatory granulocytes from mice breathing pure oxygen is in support of hyperoxic exacerbation of lung injury.

### A_2A_Rs Protect Lung Tissue from Inflammatory Damage

The genetic evidence for the critical role of A_2A_R in lung protection was provided by the observation of many more PMNs in BAL from A_2A_R-deficient mice than in BAL from similarly treated wild-type mice (Figure 5A). This was accompanied by an increase in lung vascular permeability as reflected by enhanced BAL protein levels in A_2A_R-deficient mice and a decrease in overall lung function, which was manifested by a decrease in arterial blood oxygen tension as compared to wild-type mice.

**Figure 5:**
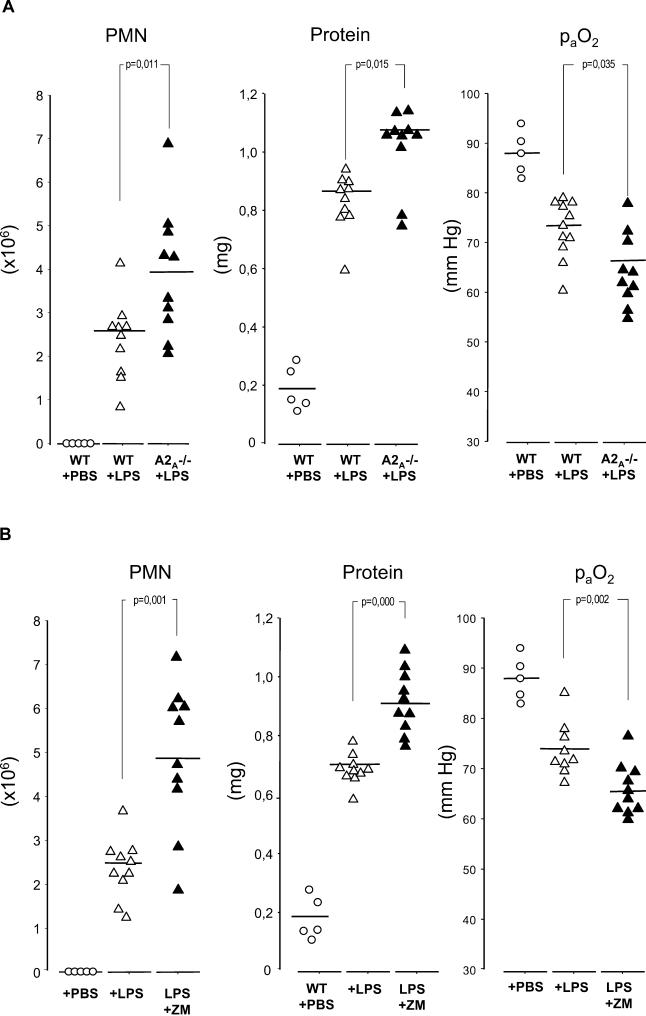
Evidence for the Critical Role of Immunosuppressive A_2A_R in Lung Protection from Inflammatory Damage (A) In *A_2A_R* gene-deficient mice, number of PMNs (left graph) and amount of protein recovered (center graph) 48 h after IT LPS injection by BAL was significantly higher than in similarly treated wild-type control mice, reflecting increased lung damage in the absence of A_2A_R. The arterial oxygen tension (right graph) was lower in *A_2A_R* gene-deficient mice as compared with wild-type mice. (B) Pharmacologic inactivation of A_2A_R leads to exacerbated inflammatory lung tissue damage and decreased lung funciton. After estimation of biologically relevant half-life of A_2A_R antagonist ZM241385 (ZM) in vivo (unpublished data), the IT LPS-injected mice were administered ZM241385 at a dose of 10 mg/kg body weight every 6 h subcutaneously to ensure sufficient levels of the antagonist. This dosing regimen of the A_2A_R antagonist caused significant more lung tissue damage, as reflected by increased number of PMNs (left graph) and protein levels (center graph) in the BAL fluid obtained after 48 h. In parallel experiments, the A_2A_R antagonist decreased lung function (right graph) as compared to untreated wild-type mice, in agreement with results of experiments with *A_2A_R* gene-deficient mice.

In agreement with the genetic evidence in Figure 5A, similar proinflammatory changes in numbers of granulocytes, levels of protein, and values of lung gas exchange were observed after pharmacological inactivation of A_2A_R with the antagonist ZM241385 (Figure 5B).

These observations establish that the A_2A_R is critical in limiting inflammatory lung injury; even higher lung injury would have resulted from the inflammatory stimuli we used, were it not for the lung protection in wild-type mice due to the inhibition of neutrophils by a functional hypoxia → A_2A_R pathway.

### Hypoxia Down-Regulates Neutrophils and Protects Lung Tissue from Inflammatory Damage

To test whether anti-inflammatory lung-protective effects can be induced in mice by allowing them to breathe a hypoxic gas mixture, LPS-injected wild-type mice were exposed to 10% oxygen. Although this oxygen concentration was sublethal for LPS-injected mice, we chose to study the effects of this degree of hypoxia because 15% of ARDS patients die from therapy-refractory hypoxemia [18]. Accordingly, some deaths occurred in IT LPS-injected mice at hour 4–7 of hypoxic exposure. In the majority of surviving mice (over 90%), however, hypoxia for 48 h strongly inhibited acute neutrophilic inflammation and led to overall better lung protection compared to mice kept at 21% oxygen. This improvement was evidenced by a decreased pulmonary sequestration of PMNs (Figure 6), inhibition of their capability to produce oxygen-reactive metabolites (Figure 6A), decreased protein accumulation (Figure 6B), and better lung function as reflected in higher arterial pO_2_ levels (Figure 6A). This conclusion was further confirmed by histological studies (Figure 6C) that revealed not only a decrease in PMN sequestration, but also a significant decrease in parameters of lung tissue damage as evidenced by the 7-fold reduction of the lung injury score (LI; Figure 6C).

**Figure 6:**
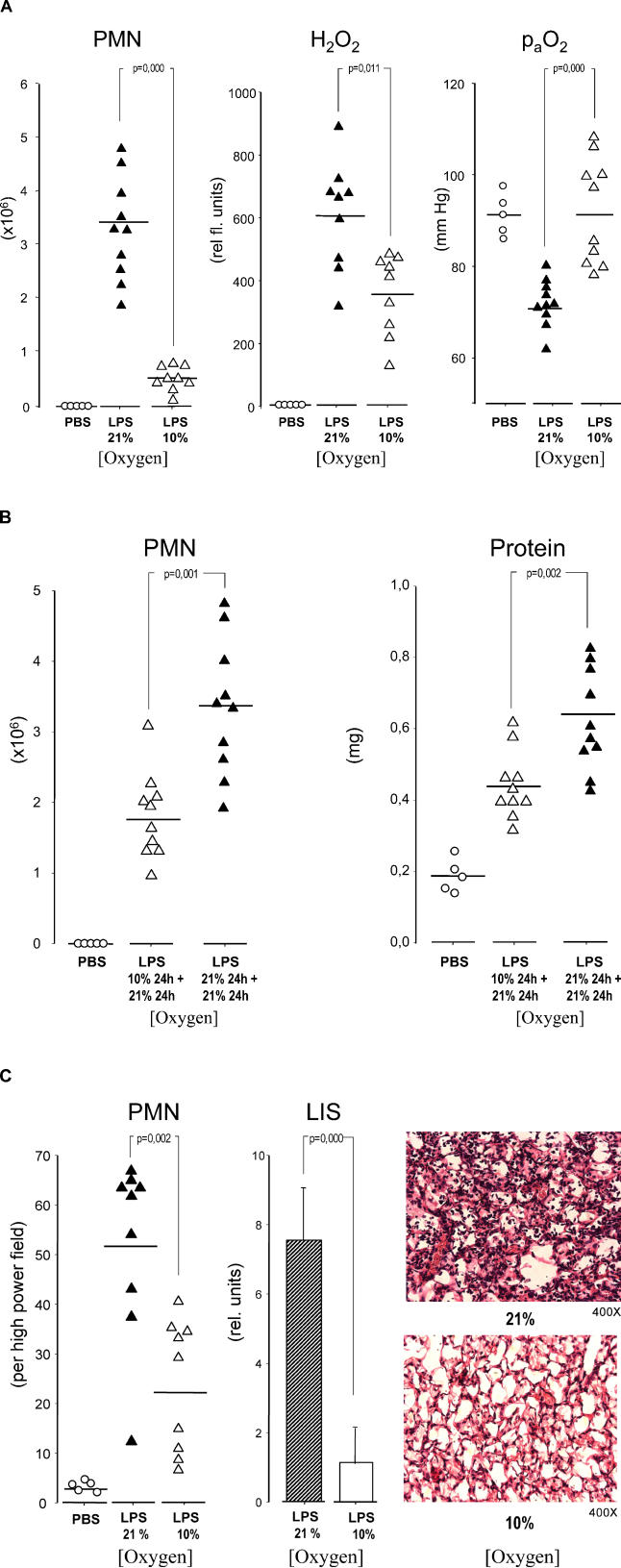
Hypoxia Down-Regulates Neutrophils and Protects Lung Tissue from Inflammatory Damage (A) Exposure of IT LPS-injected mice to hypoxic (10%) oxygen levels for 48 h atmosphere leads to a significantly decreased accumulation of PMNs (left graph), production of LPS-triggered oxygen reactive metabolites in lungs (center graph), and improved lung gas exchange (right graph) compared to a control group of endotoxin-treated mice that were kept at ambient (21%) oxygen. To standardize conditions, the arterial blood samples were taken 15 min after return of the previously hypoxia-exposed animals to normal atmosphere. (B) Treatment by a shorter period of hypoxia attenuates PMN sequestration (left graph) and lung vascular permeability (right graph). Hypoxic treatment of mice even for only 24 h was sufficient to delay PMN sequestration and to diminish the increase in lung vascular permeability. (C) Histologic evidence for the hypoxic inhibition of pulmonary PMN sequestration. Quantitative analysis of lung slices by a pathologist blinded to the experimental design revealed inhibition of PMN sequestration in IT LPS-injected mice following 4-h exposure to hypoxia. Hypoxia not only attenuated PMN accumulation, but the lung tissue damage was also significantly decreased as assessed by the LIS (*n* = 9, mean ± standard deviation). The representative H&E-stained slices in the right two photomicrographs show less intravascular granulocyte sequestration, less thickening of the alveolocapillary membrane, and almost no granulocytes in the alveolar spaces as compared to IT endotoxin-injected animals breathing 21% O_2_. These observations demonstrate that hypoxia also inhibited the transmigration of granulocytes from capillaries into the alveolar spaces.

Taken together, these data strongly suggest that tissue hypoxia is important in protecting pulmonary tissues from additional inflammatory damage.

### Hypoxia and A_2A_R-Triggered Signaling Function in the Same Lung Tissue-Protecting Pathway

It was important to establish whether inflamed and hypoxic lung tissues are protected by enhanced adenosine formation and subsequent A_2A_R engagement or by an anti-inflammatory role of a yet-to-be-uncovered hypoxia → unknown endogenous anti-inflammatory molecule “X”-mediated pathway. To distinguish between these two possibilities we tested the effects of genetic deficiency of the A_2A_R on inflammatory lung injury in hypoxic conditions.

Exposure of healthy wild-type mice to 10% oxygen resulted in a drop of arterial blood pO_2_ values to levels observed during severe hypoxemia (Figure 7A, left graph). As a result of insufficient systemic oxygen delivery and resulting hypoxemia, breathing of 10% oxygen caused an increase in production of endogenous adenosine, shown by the rise of extracellular plasma concentrations of the nucleoside (Figure 7A, right graph).

**Figure 7:**
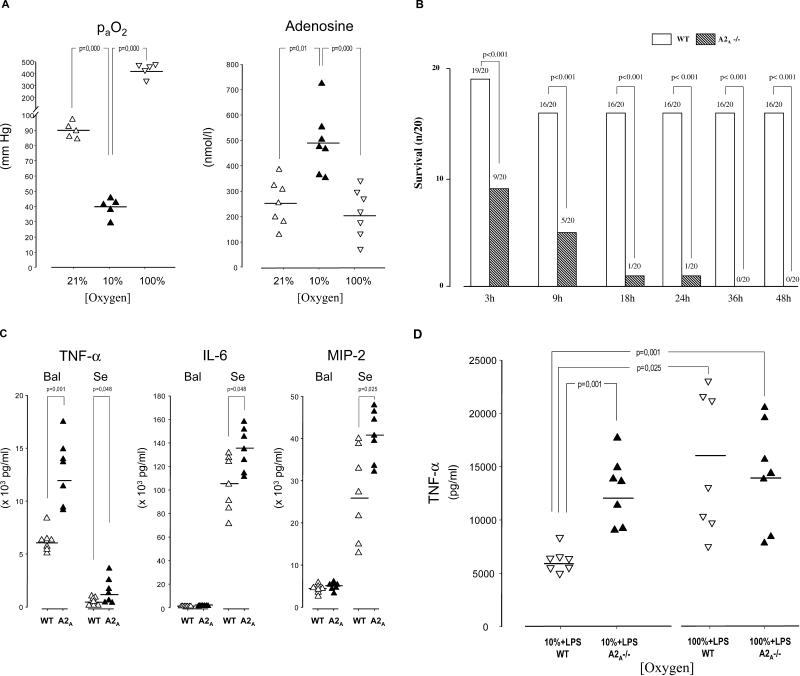
Hypoxia and Extracellular Adenosine A_2A_R Function in the Same Anti-Inflammatory, Lung Tissue-Protecting Pathway (A) Effects of breathing hypoxic (10%) oxygen on arterial blood oxygen tension (left graph) and plasma adenosine concentration (right graph) in healthy wild-type mice. As a control, data are also shown for healthy mice breathing 21% and 100% oxygen. (B) No survival of *A_2A_R* gene-deficient mice was observed in acute hypoxic lung injury. Wild-type and *A_2A_R* gene-deficient mice were injected IT with LPS and exposed to hypoxia (10%). While the majority of wild-type mice survived, all of the *A_2A_R* gene-deficient mice died, indicating that expression of A_2A_R is required for survival of hypoxic lung inflammation; this experiment mimics the clinical situation in which lung inflammation increases to such severity that hypoxia occurs. (C) Significantly higher levels of pulmonary and systemic inflammatory cytokine production in hypoxic A_2A_R-deficient mice. Observations of survival were supported by significantly higher BAL and serum (Se) levels of inflammatory cytokines in hypoxic A_2A_R-deficient mice compared to hypoxic wild-type mice. Cytokines were determined 2 h after IT LPS injection, because A_2A_R-deficient mice started to die soon after LPS administration and thus could not be used in comparative studies with wild-type control mice. The early mortality of A_2A_R-deficient mice also did not allow the comparative determination of effects of hypoxia on other late markers of inflammation such as PMN accumulation, lung vascular permeability, and pulmonary gas exchange, which in wild-type mice need about 48 h to develop after IT endotoxin injection. (D) Degree of inflammation is independent from level of oxygen in A_2A_R-deficient mice but not in wild-type mice. While BAL fluid TNF-α concentration determined 2 h after IT LPS injection was significantly suppressed in hypoxic wild-type mice compared to animals breathing 100% oxygen, hypoxia had no effect on TNF-α BAL concentrations in *A_2A_R* gene-deficient mice, demonstrating that suppression of TNF-α formation by hypoxia is mediated through A_2A_R signaling.

Hypoxic (10%) oxygen was also expected to provide an important readout of the degree of lung injury by the level of survival in the mice. In our experiments, the dose of LPS injected IT induced much higher levels of lung injury in A_2A_R-deficient mice than in control wild-type mice (see Figure 5), but neither wild-type or A_2A_R deficient mice died if they were kept at 21% oxygen at these levels of lung injury.

Different outcome with respect to death rates were expected, if A_2A_R-deficient and wild-type mice with LPS-inflamed lungs were kept at 10% oxygen. We reasoned that under these conditions the A_2A_R-deficient mice would have much more pronounced pulmonary inflammation and hence less healthy lung tissues left to adequately oxygenate vital organs. Thus, the interruption of the hypoxia → A_2A_R pathway and the resulting uninhibited inflammatory processes and increased lung damage would lead to accelerated death of A_2A_R-deficient mice in 10% oxygen.

In contrast, these treatments would not result in pronounced lethality in lung-inflamed wild-type mice at 10% oxygen tension, because wild-type mice benefit from their lung-protecting hypoxia → A_2A_R pathway and would have much larger portions of still-healthy lungs left to ensure sufficient oxygen supply to tissues.

Accordingly, we predicted that the majority of A_2A_R genetically deficient mice would die at 10% oxygen, while the majority of wild-type mice would survive. This prediction was based on the assumption that the hypoxia → A_2A_R pathway is nonredundant and would not be substituted by another hypoxia → “X” molecule receptor pathway.

These expectations were confirmed by the data showing that exposure of the IT LPS-injected wild-type mice to hypoxia resulted in low mortality (only 20% [4 out of 20] of the mice died), whereas in the parallel group all (20 out of 20) similarly treated A_2A_R-deficient mice died (Figure 7B). Control experiments with healthy, non-LPS treated, wild-type and A_2A_R-deficient mice subjected to hypoxia demonstrated that deficiency in the A_2A_R did not cause death per se, because all mice survived (unpublished data).

These observations of survival were further supported by measurements of BAL and plasma levels of the cytokines tumor necrosis factor α (TNF-α) and interleukin 6 (IL-6), and the chemokine major intrinsic protein 2α (MIP-2α), all of which are considered to be early mediators of lethal endotoxic shock [19, 20]. Accordingly, the outcome of survival experiments (Figure 7B) was in agreement with that from higher concentrations of TNF-α, IL-6, and MIP-2α in hypoxic (10% oxygen) A_2A_R-deficient mice as compared to hypoxic wild-type mice 2 h after IT LPS injection (Figure 7C). No time points after 2 h could be compared in wild-type versus A_2A_R-deficient mice, because A_2A_R knockout mice started to die soon after IT LPS-injection (Figure 7B).

Interestingly, the increase in hypoxia A_2A_R-controlled TNF-α levels was accounted for by local accumulation in the lung, because BAL concentrations of this cytokine were on average ten times higher than in the systemic circulation, although the BAL volume was at least three times higher than that of a mouse's blood. By contrast, levels of IL-6 and MIP-2α were much higher in the systemic circulation, suggesting that TNF-α BAL levels may serve as a useful marker for lung inflammation (Figure 7C). Moreover, the results shown in Figure 7D support the view that pulmonary proinflammatory cytokine TNF-α levels are indeed under the negative control of the hypoxia → A_2A_R pathway. This is supported by data showing no differences in BAL TNF-α concentrations between *A_2A_R* gene-deficient mice breathing either 10% or 100% oxygen. In contrast, the BAL TNF-α concentrations were significantly lower in hypoxic wild-type mice (with functioning hypoxia → adenosine → A_2A_R pathway) than in mice breathing pure oxygen (Figure 7D).

Taken together, these experiments support the view that both hypoxia and A_2A_R are needed to down-regulate lung inflammation, and that oxygenation exacerbates lung injury due to interruption of the tissue-protecting hypoxia → A_2A_R anti-inflammatory pathway. Thus, these observations leave no room for yet another lung tissue-protecting hypoxia → X receptor signaling pathway in tested experimental conditions. This knowledge suggested a direct and effective therapeutic countermeasure to reap benefits of oxygenation without sacrificing still-healthy lung tissues to continuing inflammatory damage: use of the A_2A_R agonist CGS21680.

### An A_2A_R Agonist Compensates for the Loss of Lung-Protective Mechanisms and Prevents Death

Figure 8 shows that IT injections of the selective A_2A_R agonist CGS21680 significantly inhibited lung injury in LPS-treated mice. This treatment led to (i) significantly decreased accumulation of PMNs (Figure 8A), (ii) reduced production of reactive oxygen metabolites (Figure 8A), (iii) less-pronounced increases in lung vascular permeability (Figure 8B), and (iv) improved lung gas exchange (Figure 8B). Histological examination of CGS21680-treated mice revealed that therapeutic effects of the agonist were similar to those of exposure of mice to hypoxia. CGS21680 treatment resulted in inhibition of pulmonary PMN sequestration (Figure 8C), and—as shown for hypoxia above—was followed by a significant reduction of lung tissue damage as assessed by the 4-fold decrease in the LIS (Figure 8C).

**Figure 8:**
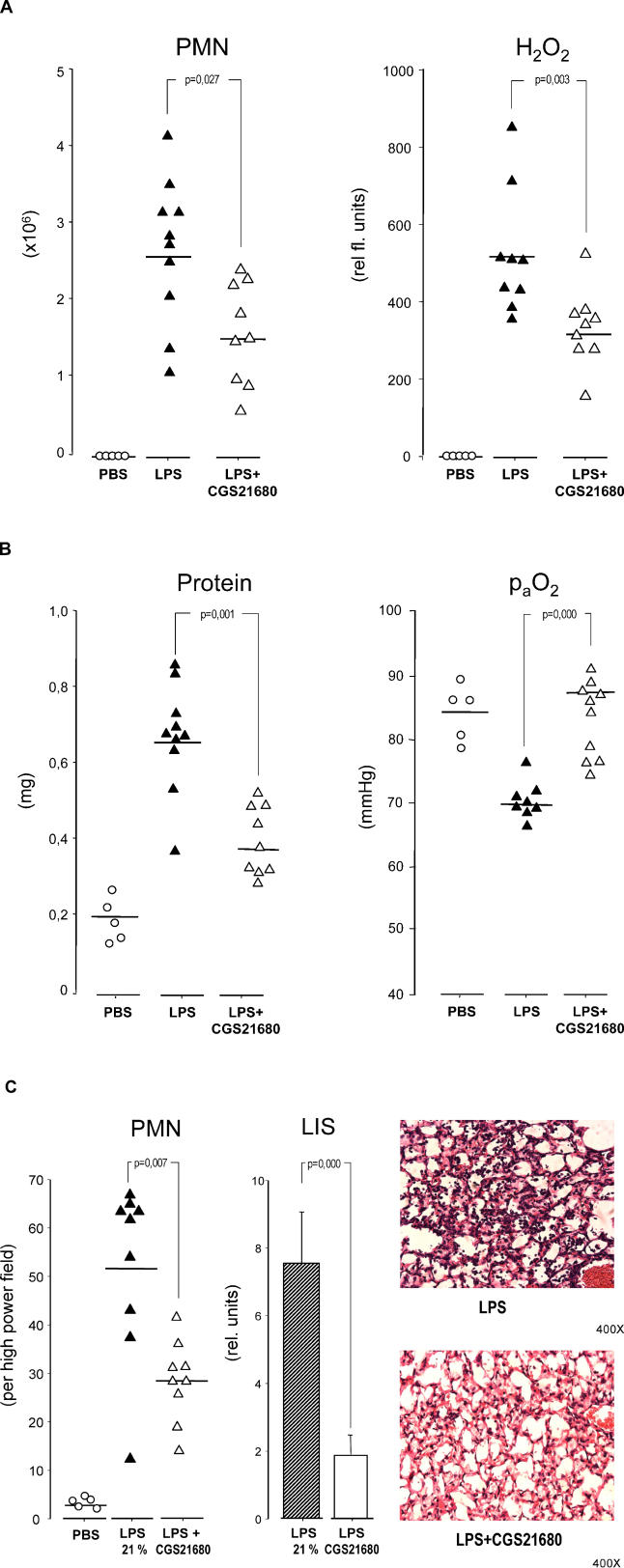
Intratracheal Injection of A_2A_R Selective Agonist Mimics Protective Effects of Hypoxia (A) IT injection of the A_2A_R agonist CGS21680 into endotoxin-inflamed lungs provides protection similar to that observed in hypoxia-treated mice. Number of PMNs recovered after 48 h by BAL from endotoxin-injected animals that were kept at normal 21% oxygen atmosphere was significantly diminished by IT injections of CGS21680 compared to placebo-treated mice. Lung PMNs (left graph) from A_2A_R agonist-treated animals also produced lower levels of reactive oxygen metabolites (H_2_O_2_; right graph). (B) Significantly decreased lung vascular permeability (protein in BAL; left graph) and improved lung gas exchange (p_a_O_2_; right graph) in endotoxin-injected mice after treatment with the A_2A_R agonist CGS21680. (C) Histologic evidence for the lung tissue-protecting effects of A_2A_R agonist during endotoxin- and oxygenation-induced lung damage. Quantitative analysis of lung histopathology by a pathologist blinded to the experimental design revealed inhibition of PMN sequestration in IT LPS-injected mice after treatment with the A_2A_R-selective agonist CGS21680 for 48 h. The lung tissue damage was also significantly decreased as assessed by the LIS (*n* = 9, mean ± standard deviation). Representative H&E-stained slices in the right two photomicrographs show less intracapillary PMN sequestration and almost no intraalveolar accumulation of PMNs in CGS21680-treated mice. These CGS21680-induced changes are similar to those observed for the effects of hypoxia on endotoxin- injected animals (compare with Figure 6C).

Treatment with CGS21680 was effective even when applied in the more severe polymicrobial toxin model of lung injury (i.e., LPS + SEB); IT injections of this agonist under hyperoxic conditions rescued the majority of mice from oxygenation-induced death. The death rate was 80% among oxygenated mice with inflammatory lung injury in the control group, but dramatically less among those treated with the A_2A_R agonist (Figure 9).

**Figure 9:**
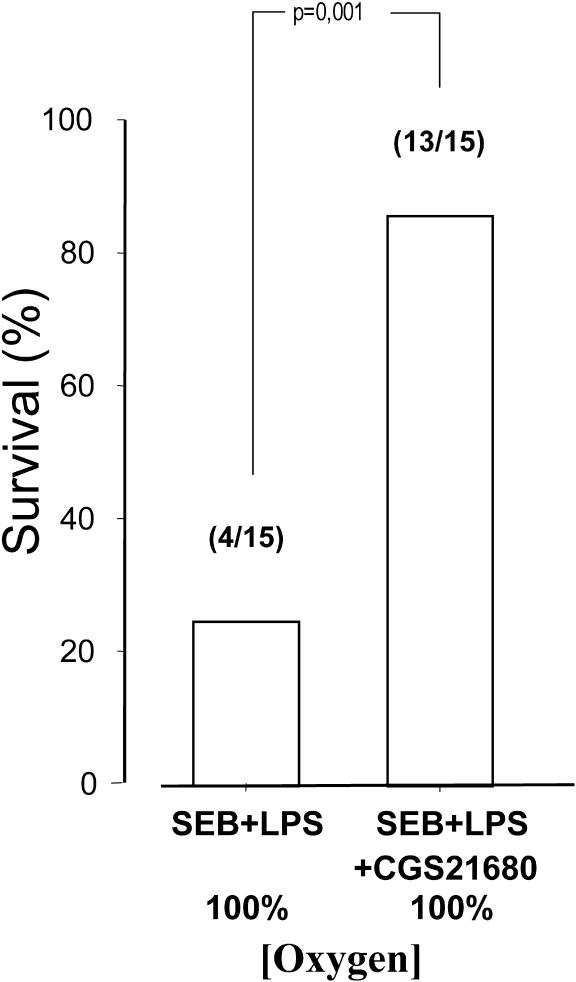
IT Administration of A_2A_R Agonist Protects from Increased Death Rate upon Oxygenation of Mice with Acute Lung Injury Compensation for the oxygenation-associated loss of the hypoxia → adenosine → A_2A_R signaling pathway by IT injection of CGS21680 significantly decreased the oxygen-exacerbated death rate in mice with acute lung injury induced by IT injection of SEB and LPS. For further explanation, see legend for Figure 1.

Thus, the exogenously added, selective, synthetic A_2A_R agonist compensated for the loss of endogenously formed adenosine in oxygenated inflamed lungs, thereby decreasing lung injury and rescuing mice from death. In an important control, CGS21680 at the dosing regimen used to treat wild-type mice was proven to be selective, since it did not affect lung inflammation in *A_2A_R* gene-deficient mice (unpublished data).

## Discussion

The oxygenation of hypoxic patients with impaired lung function is an important and life-saving therapeutic measure, but 15% of patients with ARDS still die from treatment-refractory hypoxia [18]. We here provide evidence supporting the hypothesis that, in a mouse model of lung inflammation, while oxygenation relieves the immediate life-threatening consequences of hypoxemia, it also further exacerbates acute lung injury and even may lead to death due to the interruption of the critically important, nonredundant hypoxia → adenosine → A_2A_R-mediated lung-protecting pathway. This conclusion is supported by (i) the strong increase in lung inflammation and mortality after short-term exposure of mice to high and even to moderately elevated concentrations of oxygen (see Figures 1 and 2); (ii) the causative pathogenic role of PMNs in inflammatory lung injury (see Figure 3) and the up-regulation of A_2A_R expression on alveolar PMNs (see Figures 4); (iii) the critical role of A_2A_R in lung protection from enhanced accumulation of PMNs as well as more pronounced vascular permeability and stronger impairment of lung gas exchange in A_2A_R genetically deficient mice (see Figure 5A) and in A_2A_R antagonist-treated wild-type mice (see Figure 5B); (iv) the strong lung-protective effects of hypoxia by suppression of PMN emigration, PMN activation, lung vascular permeability, and impairment of gas exchange in wild-type mice (see Figure 6); (v) the evidence that hypoxia is upstream of A_2A_Rs in the same anti-inflammatory, lung tissue-protecting pathway, as shown by the failure of the adenosine-producing (see Figure 7A) hypoxia to protect A_2A_R-deficient mice from death and excessive pulmonary TNF-α cytokine production (see Figure 7B–7D), and (vi) the confirmation of the hypoxia-elicited lung-protective effects in wild-type mice by the A_2A_R agonist CGS21680 (see Figure 8), which (vii) could rescue mice from hyperoxia-accelerated death (Figure 9).

By confirming the molecular mechanism of the predicted exacerbation of lung injury by oxygenation, this mechanism also offers a direct and effective preventive measure by compensating for the oxygenation-related loss of endogenous adenosine. Accordingly, we suggest the reevaluation of relevant ventilation and oxygenation protocols and the consideration of compensatory therapeutic treatments of oxygenated-inflamed lungs with A_2A_R agonists (reviewed in [7]) to prevent uninterrupted inflammatory lung damage (see Figure 8) and death (Figure 9). Other anti-inflammatory drugs alone or in combination with an A_2A_R agonist should be also considered in future refinements of this approach, because an A_2A_R agonist offers the advantage of pharmacologically restoring the physiological tissue-protecting pathway [4, 11, 12], which is unintentionally weakened by therapeutic oxygenation.

It is likely that these findings are most applicable to ARDS patients, although other clinical situations in which inflamed lungs are oxygenated should be also considered. The lungs of ARDS patients are known to be heterogenously ventilated, leaving substantial lung areas hypoxic with up to 33% of total lung volume being nonaerated [21]. These are the most damaged and therefore hypoxic lung regions, where—counterintuitively—hypoxia may protect still-healthy surrounding lung tissues from the additional inflammatory injury by promoting formation of extracellular adenosine [8–10] and strengthening the anti-inflammatory A_2A_R signaling pathway [4–7, 11, 12]. Supportive ventilation to increase lung tissue oxygen levels also eliminates the hypoxia-associated formation of anti-inflammatory endogenous adenosine, and this allows the unopposed continuation of the inflamed lung destruction. Thus, a necessary medical intervention (oxygenation of oxygen-deprived patients) may also cause an iatrogenic exacerbation of the very condition that led to the need for oxygenation in the first place. Such pathophysiological consequences of tissue oxygenation are expected to be most pronounced in collapsed hypoxic lung areas that would be recruited by any kind of ventilatory strategy [22].

In agreement with our findings are sporadic observations of effects of oxygenation in other clinical human protocols [23, 24] and experimental assays in animal models [25, 26]. For instance, intraoperative administration of 100% oxygen augmented proinflammatory cytokine production of alveolar macrophages within 2–8 h of the start of anesthesia and surgery in patients [23, 24]. Similarly, in animal models of lung injury, a synergistic action between infectious agents [25], bacterial toxins [26], or acid aspiration [27] and hyperoxia was demonstrated and difficult to explain by direct oxygen toxicity. Indeed, the time periods of these effects were much too short for the manifestation of oxygen toxicity, which usually takes more than 64–72 h to become clinically apparent in mice [14]. Of note, in the same mouse strain (C57BL/6) that we used in our studies, breathing of pure oxygen did not result in death before the fourth day [15].

Although it is likely that synergy between lung-damaging noxious agents and oxygen is mediated by oxygen radicals [27], the elimination by oxygenation of the natural anti-inflammatory hypoxia → A_2A_R signaling pathway was not appreciated before, which may account for a significant proportion of inflammatory complications in patients.

Our observations of potent anti-inflammatory effects of hypoxia in lung injury (see Figures 6 and 7D) are in agreement with previously published effects of hypoxia, including significant attenuation of emigration of neutrophils to the site of inflammation in carrageenin-induced pleurisy [28], inhibition of granulocyte adhesion to endothelial cells [29], enhanced shedding of adhesion molecule CD11b from PMNs [30], and suppression of cytokine formation when PMNs are in contact with extracellular matrix proteins [31]. Use of *cd39*- and *cd73*-null animals revealed that extracellular adenosine produced through adenine nucleotide metabolism during hypoxia is a potent mechanism attenuating excessive tissue PMN accumulation [9]. Hypoxia was also shown to strongly inhibit production of MCP-1 [32], IL-1β [33], granulocyte-macrophage colony-stimulating factor [34], and induced down-regulation of cosignaling molecules (CD80) [35]. Hypoxia was further shown to cause decreased expression of Toll-like receptor 4 receptors by inhibiting translocation of activator protein 1 [36] and caused suppression of Escherichia coli-induced nuclear factor κB and activator protein 1 transactivation [37]. Finally, hypoxia was shown to stimulate phosphatidylinositol 3-kinase activity and thereby protect human lung microvascular endothelial cells and epithelial type II-like A549 cells from subsequent oxygen toxicity [38].

However, hypoxia was also shown to exert some proinflammatory effects in vitro [39] and in vivo [9, 40, 41] for time intervals ranging from 3 to 12 h, which may not reflect the long-term effects of hypoxia on inflammatory processes as assessed in our study.

Importantly, the virtually complete lung protection from the proinflammatory effects of 100% oxygen by an immunosuppressive A_2A_R agonist (Figure 9) supports the view that exacerbation of lung damage by 100% oxygen could be almost fully accounted for by immune mechanisms in addition to mechanical [42,43] or radical-mediated mechanisms of lung injury [2]. It remains to be determined, however, whether the much longer time course of the direct toxicity of 100% oxygen in healthy, noninflamed lungs could be explained by the recruitment of immune cells [44], which then, in turn, would exacerbate the 100% oxygen-triggered lung tissue damage through prolongation of trauma-initiated inflammation.

Finally, the observations and conclusions of this report may have implications for the widely used therapeutic oxygenation in patients with lung injury, and may answer the long-standing question as to why lungs are the most susceptible to inflammatory injury and why lung failure usually precedes multiple organ failure [45, 46]. We propose that the diseased lung might be uniquely susceptible to iatrogenic damage by oxygenation, because the lungs are exposed to the highest levels of oxygen tension of all organs. Therefore, this critical, A_2A_R signaling-mediated, hypoxia-driven, lung tissue-protecting anti-inflammatory mechanism is the most vulnerable to elimination by oxygenation in the hypoxic areas of damaged lung as compared to other vital organs.

## Materials and Methods

### 

#### Mice

Female 6- to 8-wk-old C57BL/6 *A_2A_R* gene-deficient mice (N10) and age-matched wild-type mice were maintained in pathogen-free NIH animal facilities. The *A_2A_R* genotypes of mice were determined by Southern blot analysis as described previously [47]. All animal study protocols were approved by the NIH Animal Care and Use Committee.

#### IT administration of LPS and SEB.

Female C57BL/6 mice were anesthetized by isoflurane anesthesia. Using a modified transtracheal illumination technique [48], mice were nontraumatically intubated by intratracheal insertion of a 24-gauge catheter (Abbocath-T, Abbott Ireland, Ireland) via direct laryngoscopy. SEB was injected IT at a dose of 1 mg/kg body weight in a total volume of 50 μl per mouse. After IT injection of unconscious but spontaneously breathing mice, animals were held in an upright position for 15 s and then briefly shaken in all directions to ensure homogenous fluid dispersion in the lung. After 1 h, mice were anesthetized again with isoflurane and injected with LPS at a dose of 4 mg/kg body weight in a total volume of 50 μl per mouse. The injected fluid was dispersed by the same positioning maneuver as applied for the intrapulmonary distribution of SEB. Control, sham-treated mice were intubated and injected with only the solvent that was used to inject toxins, i.e., phosphate-buffered saline.

#### LPS administration

LPS was IT injected at a dose of 2 mg/kg body weight in a total volume of 50 μl per mouse under isoflurane anesthesia using the same technique as described above.

#### Depletion of granulocytes in mice.

Mice were depleted in granulocytes by two IV injections of anti-GR-1 monoclonal antibody at a dose of 1 mg/kg body weight, administered 24 h before and briefly before IT injection of LPS. Control animals received isotype-matched antibody at the same dose and intervals.

#### In vivo administration of A_2A_ receptor agonist and antagonist.

The A_2A_R agonist CGS21680 was dissolved in PBS and administered by IT injection at 0,1 mg/kg body weight in a total volume of 50 μl per mouse. IT injection of CGS21680 was repeated every 8 h until termination of the experiment. In control animals, the solvent of CGS21680, i.e., PBS only, was administered in the same way and on the same schedule. CGS21680 solution or solvent were injected within 15 min of administration of SEB and/or LPS in both models of lung injury. In studies using ZM241385, the A_2A_R antagonist was injected subcutaneously at a dose of 10 mg/kg body weight every 6 h until the end of the experiment. ZM241385 was dissolved in DMSO (10 mg/145 μl of DMSO) and then further diluted in 14,8 ml of PBS, yielding a 2 mM working concentration of which a volume of approximately 250–300 μl was injected per mouse. Control animals received the same volume of the solvents only. ZM214385 or control solution were injected subcutaneously 30 min before IT LPS administration. According to the pharmacokinetics of subcutaneously injected ZM214385 at 10 mg/kg body weight, plasma concentrations of the antagonist were higher than 50 nM, even 6 h after its administration. This concentration of ZM241385 is sufficient to exert maximum pharmacologic antagonism on the adenosine-induced cAMP response in murine thymocytes (for determination of pharmacokinetics of ZM214385 see also “Modulation and measurement of cAMP production of PMNs by A_2A_R stimulation and antagonism in vitro,” below).

#### Control of fraction of inspired oxygen.

In order to modify the fraction of inspired oxygen, mice were placed in airtight modular incubation chambers (Billups-Rothenberg, San Diego, California, United States), and the atmosphere was controlled by a constant gas flow (1,5 l/min) of desired composition (10% O_2_, 60% O_2_, or 100% O_2_). To prevent any CO_2_ retention in the chambers, the chambers' bottom was covered with approximately 250 g of anesthetic CO_2_ absorber material (Sodasorb, Grace & Co, Chicago, Illinois, United States). Composition of gas atmosphere was tested intermittently by analyzing pCO_2_ and pO_2_ values in an equilibrated fluid sample drawn from a tube inside the chamber whenever the latter was opened for reinjection of mice. During the stay of mice in chambers, animals were fed and given fluids by offering them Transgel (Charles River Laboratories, Wilmington, Massachusetts, United States).

#### Assessment of lung gas exchange and oxygen tension in peripheral blood.

At the end of the experiments, arterial blood specimen from the tail artery were sampled directly into heparinized glass capillaries that were closed on both ends with Parafilm and kept on ice until further analysis (Rapidlab 248 system; Chiron Diagnostics, Essex, United Kingdom).

#### Histological analysis.

IT LPS-injected mice were sacrificed by isoflurane anesthesia, and the trachea was surgically exposed and cannulated with a 20-gauge needle. The thoracic cage was opened to allow lungs to expand during injection of 1 ml of 4% paraformaldehyde. After 15 min, the lungs were removed and fixed in 4% paraformaldehyde until processing and H&E staining (American Histolabs, Washington, Maryland). Quantitative analysis of lung neutrophil sequestration was performed as previously described [49], and lung tissue injury was semiquantitatively assessed in a blinded fashion by a professional pathologist (I. Bittmann, MD). Lung slices of IT LPS-injected groups of mice were first assessed for pathological changes in general, enabling identification of lung inflammation parameters to be further evaluated in a semiquantitative way by assignment of four degrees of increasing severity. Overall assessment of slides showed acute blood congestion, intraseptal and intraalveolar neutrophil sequestration, interstitial edema, intraalveolar macrophage accumulation, pneumocyte type II activation, endothelial cell activation, and endothelial adherence of leukocytes. No intraalveolar edema and no fibrin deposits were observed. Lung tissue injury was then evaluated by assignment of four degrees of severity (0–3) to the following parameters in H&E stained lung slides: interstitial edema formation, intraalveolar macrophage accumulation, pneumocyte activation, endothelial cell activation, and leukocyte adhesion to endothelial cells. The lung injury score of each lung slice was calculated as an average of each parameter's degree of severity, ranging from 0 for a healthy lung to 15 (= 5 × 3) for a maximally injured lung. Further details on definition of degrees of severity of pathological changes of each parameter are described in Table 1.

**Table 1:**
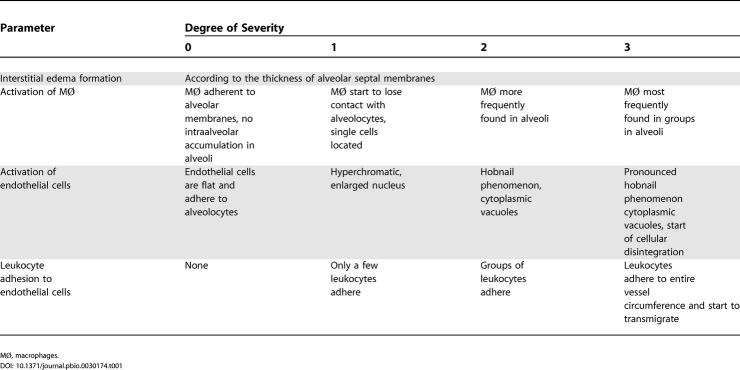
Determination of Lung Injury Score

MØ, macrophages.

#### BAL and cellular differentials.

Following sacrifice and surgical preparation of mice as outlined above for histological analysis, except the opening of the thoracic cavity, lungs were lavaged by injection of 0,6 ml of ice-cold HBSS, which after each collection was repeated four times. The recovered (over 90%) BAL fluid was processed for total cell counts and cellular differentials. Contaminating erythrocytes were lysed by 0,01% saponin, and total leukocytes counted with a Neubauer chamber. Differential counts were performed after cytospin onto glass slides and staining with Hema-3 stain. The remaining bronchoalveolar fluid was spun down and the supernatant collected for determination of protein concentration using the Bio-Rad Protein Assay (Bio-Rad, Hercules, California, United States) according to the manufacturer's manual. The cell pellet was used for flow cytometric detection of hydrogen peroxide production by neutrophils.

#### Flow cytometric determination of hydrogen peroxide production by activated PMNs.

BAL cells or whole blood, the latter withdrawn from the tail vein and diluted in ice-cold heparinized (40 IE/ml) HBSS, were washed two times with HBSS (0 °C). Cell pellets were resuspended in HBSS containing dihydrorhodamine (2,5 μM). For measurement of spontaneous hydrogen peroxide production by oxidation of dihydrorhodamine to fluorescent rhodamine, cells were either left on ice or incubated at 37 °C for 30 min, then returned thereafter to ice. In parallel experiments designed to test the effects of CGS21680 on blood granulocytes, cells were preincubated with LPS (10 μg/ml) in the absence or presence of the A_2A_R agonist for 15 min, then stimulated by addition of the chemotactic tripeptide fMLP (10 μM). When pharmacologic effects of CGS21680 were tested on BAL granulocytes, no LPS was added. Cells were washed two times with ice-cold PBS that contained bovine serum albumin (0,5%). FcgII/III receptors were blocked by anti-mouse CD16/CD32 (10 min, 1 mg/ml) and cells were incubated with PE-anti-mouse Ly6G (Gr-1) (15 min, 0,5 mg/ml). After washing with PBS, cells were subjected to flow cytometry. Granulocytes were identified by staining for Gr-1 on Fl-2. Hydrogen peroxide production was determined by the fluorescence intensity of rhodamine on Fl-1. Appropriate compensation was set on fluorescence channels to avoid signal overlap. All data were collected in the log-amplified mode, and readings linearized using CellQuest software (Becton Dickinson, San Diego, California, United States). Spontaneous activity was calculated by subtraction of values of cells kept on ice from those incubated at body temperature. Chemotactically induced activity was determined by subtraction of values obtained at body temperature from those after addition of fMLP.

#### Preparation of granulocytes from bone marrow.

Bone marrow was removed from femur and tibia by injection of HBSS medium, washed two times, and resuspended in HBSS medium. Remaining red blood cells were removed by dextran sedimentation, and neutrophils were recovered by discontinuous Ficoll density gradient centrifugation. Purity of cells (over 90% granulocytes) was checked and further used for cAMP and adenosine receptor mRNA determination as outlined below.

#### Modulation and measurement of cAMP production of PMNs by A_2A_R stimulation and antagonism in vitro

In vivo-activated granulocytes recovered by BAL from inflamed lungs or naïve granulocytes obtained from bone marrow of healthy mice were incubated in the presence of rolipram (1 μM, 37 °C, 30 min) and activated with fMLP (10 μM) in the absence and presence of increasing concentrations of the A_2A_R agonist CGS21680. Specificity was tested by either pharmacological antagonism (A_2A_R antagonist ZM241385 1 μM) or using cells from A_2A_R^−/−^ mice. After 2 min of chemotactic activation, cAMP metabolism was stopped by addition of HCl and putting cells on dry ice. Cell suspensions were kept frozen (−80 °C) until analysis of cAMP levels as previously described [50]. Freshly prepared thymocytes from wild-type mice in which cAMP production was induced by CGS21690 (10 μM) in the absence or presence of ZM241385 were used as a positive control. Since ZM241385 concentration-dependently decreased the cAMP production of thymocyte elicited by A_2A_R activation with a fixed concentration of adenosine, the system was also used to determine the plasma concentrations of ZM241385 during the in vivo treatment of mice with the antagonist (unpublished data).

#### Determination of A_1_R- and A_2A_R-specific mRNA levels in PMNs.

Quantification of A1- and A_2A_R-specific mRNA levels were determined in naïve or in vivo-activated granulocytes according to the method published previously [51].

#### Blood sampling and determination of plasma concentrations of adenosine.

Arterial blood samples were drawn from anesthetized mice by cardiac puncture using ice-cooled syringes pre-filled with a stop solution containing EHNA (20 μM), dipyridamole (200 μM), EDTA-Na_2_ (20 mM), EGTA (20 mM), and dl-α-glycerophosphate to prevent degradation or additional formation of plasma adenosine. Mice were breathing 21% oxygen or 10% oxygen atmosphere at least 5 min before cardiac blood sampling. By obtaining arterial blood samples from the left ventricle, the loss of the adenosine due to its rapid degradation by adenosine deaminases was minimized and plasma concentrations of adenosine better reflected those in lung tissue, although local levels of adenosine in hypoxic tissues are expected to be much higher. Drawn samples were processed and plasma concentrations of adenosine determined by HPLC as previously described [52].

#### Reagents

SEB was obtained from Toxin Technology (Sarasota, Florida, United States). LPS (E. coli E055:B5) and HBSS were purchased from Sigma Chemicals (St. Louis, Missouri, United States) and supplemented by 1 mM MgCl_2_ and 1 mM CaCl_2_ freshly before use. Dihydrorhodamine was purchased from Molecular Probes (Eugene, Oregon, United States). PE-labeled anti-mouse Ly6G (Gr-1) antibody and isotype control antibody were obtained from Caltag Laboratories (Burlingame, California, United States). Fc-receptor-blocking anti-CD16/CD32 antibodies were purchased from BD Biosciences Pharmingen (San Diego, California, United States). Hema 3 stain for white blood cell differential counts was purchased from Fisher Scientific (Swedesboro, New Jersey, United States). Dye reagent concentrate for protein determination was obtained from Bio-Rad Laboratories. A_2A_R-selective agonist CGS21680 and antagonist ZM241385 were purchased from Tocris (Ellisville, Missouri, United States). A kit for cAMP enzyme immunoassay was purchased from Amersham Pharmacia Biotech (Piscataway, New Jersey, United States). Isoflurane was obtained from Baxter (Deerfield, Illinois, United States). All other chemicals were purchased from Sigma Chemicals.

#### Statistics.

Data are represented as individual values. In the graphs, the horizontal lines give the means of the individual values. Comparison between independent samples was performed by two-tailed nonparametric Mann-Whitney test. To test for the strength of the relationship between two variables, linear regression analysis was performed and Pearson correlation coefficients calculated. Differences between survival rates were tested by Chi^2^ test.

## Abbreviations

A_2A_R: adenosine A_2A_ receptor
ARDS: acute respiratory distress syndrome
BAL: bronchoalveolar lavage
cAMP: cyclic adenosine 3′,5′-monophosphate
fMLP: N-formyl-methionyl-leucyl-phenylalanine
IT: intratracheal(ly)
LIS: lung injury score
LPS: lipopolysaccharide
MIP-2α: major intrinsic protein 2α
PMN: polymorphonuclear leukocyte
pO_2_: oxygen partial pressure
SEB: staphylococcal enterotoxin B
TNF: tumor necrosis factor
